# Attachment and maternal sensitivity are related to infants’ monitoring of animated social interactions

**DOI:** 10.1002/brb3.410

**Published:** 2015-10-21

**Authors:** Szilvia Biro, Lenneke R. A. Alink, Renske Huffmeijer, Marian J. Bakermans‐Kranenburg, Marinus H. van IJzendoorn

**Affiliations:** ^1^ Center for Child and Family StudiesLeiden UniversityLeidenThe Netherlands; ^2^ Leiden Institute for Brain and CognitionLeidenThe Netherlands

**Keywords:** Attachment, eye‐tracking, infant, maternal sensitivity, social cognition

## Abstract

**Background:**

Infants have been shown to possess remarkable competencies in social understanding. Little is known, however, about the interplay between the quality of infants’ social‐emotional experiences with their caregivers and social‐cognitive processes in infancy.

**Method:**

Using eye‐tracking we investigated the relation of infant attachment quality and maternal sensitivity with 12‐month‐old infants’ monitoring patterns during the observation of abstractly depicted interactions of a “parent” and a “baby” figure.

**Results:**

We found that secure infants focused their attention on the “parent” figure relative to the “baby” figure more than insecure infants when the two figures got separated. Infants with more sensitive mothers focused their attention more on the ongoing behavior of the “parent” figure after the separation than infants with less sensitive mothers when distress of the “baby” figure was implied by accompanying baby crying sounds.

**Conclusion:**

Our findings support the notion that early social‐emotional experiences with the caregiver are related to social information processing and that these social information processing patterns might be markers of infants’ developing internal working models of attachment.

## Introduction

Infants are equipped with an inborn behavioral system that functions to form attachment relationships with their caregivers (Bowlby 1969). This system enables securely attached infants to maintain proximity to their caregivers in stressful situations and to use their caregivers as a “secure base” to explore from and to return to. Insecure infants tend to be more reluctant to rely on their caregivers to ease their stress. A large body of evidence shows that the quality of infant‐caregiver interactions (sensitivity and responsiveness of the caregiver) during the first few months has an important influence on the quality of the attachment relationship (De Wolff and Van IJzendoorn 1997; Bakermans‐Kranenburg et al. 2003). In turn, early attachment security has a significant impact on self‐ and emotion‐regulation skills later in life (Sroufe et al. 2005; Fearon et al. 2010; Groh et al. 2012).

Importantly, Bowlby and others also hypothesized that early attachment‐related experiences with the primary caregiver lead to the formation of “internal working models” about the social world, that is, in cognitive terms, mental representations or prototypes of social relations (Waters and Waters 2006; Bretherton and Munholland 2008). Expectations about one's own and others’ behavior concerning the ways in which attachment‐related events are supposed to unfold are the core properties of these representations. According to Bowlby, internal working models already start to develop in infancy and individual differences in the content or organization of the working models emerge in the first 5 years after birth. The quality of early social‐emotional experiences with the caregiver is thus assumed to bias the way in which individuals process social information and social relations outside of the parental relationship.

This hypothesis is supported by a large body of evidence showing a relation between early attachment security and the processing of certain social‐cognitive information in children, adolescents, and adults in the domains of attention and memory processes, social attributions, understanding mental states and emotions (see Dykas and Cassidy 2011 for a comprehensive review). These studies found that compared to secure individuals, insecure individuals typically either show an increased tendency to suppress the processing of attachment‐related information (e.g., they look away more from drawings depicting social interactions, show poorer memory for attachment‐related events, and are less accurate in mental state attributions) or show a negatively biased processing (e.g., they attend to emotionally or socially negative information more and faster than positive ones, remember negative information better and they tend to attribute or expect more negative intentions; Main et al. 1985; Bretherton et al. 1990; Belsky et al. 1996; Cassidy et al. 2003; Feeney & Cassidy, 2003; Maier et al. 2005; Fraley et al. 2006; Dewitte et al. 2007; Bretherton and Munholland 2008; Atkinson et al. 2009; Mikulincer et al. 2009; Dykas et al. 2010).

Recent research using novel experimental paradigms and techniques led to the discovery of early competencies in infants’ social understanding. Before their first birthday, infants already have a rather sophisticated understanding of the behavior of others. Infants can infer goals, intentions, and beliefs (e.g., Woodward 1998; Onishi and Baillargeon 2005; Southgate et al. 2010; Biro 2013), they can make predictions about social interactions that involve collaboration, helping, hindering, or sharing of resources (Kuhlmeier et al. 2003; Hamlin et al. 2007; Warneken and Tomasello 2007; Hamlin and Wynn 2010; Dunfield et al. 2011; Henderson and Woodward 2011; Sloane et al. 2012; Choi and Luo 2015). These discoveries now allow researchers to investigate the relation between the quality of early social‐emotional experiences and social information processing already in infancy. It has been argued that such studies would fill a gap in attachment theory and research (Cassidy et al. 2013).

Pioneering work by Susan Johnson and her colleagues (2007, 2010) showed that attachment security is reflected in 12‐month‐old infants’ expectations about the outcome of an abstract, animated social interaction. By measuring infants’ looking time in a violation of expectation paradigm, Johnson et al. found that only securely attached infants looked longer at a test event that showed unresponsive caregiving behavior compared to responsive caregiving. Unresponsive caregiving apparently violated secure, but not insecure, infants’ expectations. These findings provide the first experimental evidence for the presence of “internal working models” in infants and their influence on infants’ expectations of social situations that are outside the realm of their relationship with their own primary caregivers.

As a next step, the aim of our study is to investigate the nature of individual differences in infants’ social information processing biases by looking at infants’ monitoring strategies during the observation of social interactions between others. Monitoring patterns can give us a real‐time insight into infants’ allocation of attention to specific aspects of ongoing interactions, and can thus tell us about the process of infants’ information pickup. Monitoring measures, such as fixation duration at a given location, can indicate (cognitive) saliency of a region (Henderson et al. 2007) and the processing load required to retrospectively or prospectively interpret a specific aspect of a scenario (Klein et al. 2009; Zwickel et al. 2010). Monitoring can be affected by previous knowledge and expectations (Hayhoe et al. 2003; Biro et al. 2014a; Biro et al. 2014b) and also by perceptual properties of the observed scenario (Tellinghuisen et al. 1999; Parkhurst et al. 2002). Using eye‐tracking methodology with infants, monitoring measures have for example been found to be sensitive indicators of the familiarity or the threat‐relatedness of facial expressions (Peltola et al. 2009; Hunnius et al. 2011; Gredebäck et al. 2012). Furthermore, risk for autism and depression of the caregiver have also been related to individual differences in the monitoring of faces and facial expressions (Striano et al. 2002; Merin et al. 2007). Most relevantly, Peltola and his colleagues (2015) recently showed that attachment insecurity, particularly with increasing signs of disorganized attachment, is associated with the size of attentional bias to fearful faces. Very little is known, however, about the influence of attachment quality and caregiving environment on infants’ monitoring strategies of others’ social interactions.

In our previous eye‐tracking study with a normally developing infant sample (Biro et al. 2014b), we tested the influence of emotional signals on the monitoring patterns of infants who watched animations depicting the interaction of two abstract characters (similar to the ones used in the Johnson et al.'s study; 2007). We found that when infants heard the sound of a crying baby during the separation of the characters, they focused their attention on the “parent” character more than when a laughing baby sound was heard. However, the type of emotional signal did not alter the monitoring of the “baby” figure. We suggested that this character‐specific monitoring difference between the animations with different emotional signals was due to the fact that the separation accompanied by a crying sound indicated an unresolved distress situation in which more cognitive processing was required to understand the role of the “parent” figure (retrospective interpretation) and/or to anticipate a reaction from the “parent” figure to solve the situation (prospective interpretation) than in the laughter scenario.

Motivated by Johnson et al.'s (2007, 2010) and our previous findings (Biro et al. 2014b), this study investigates whether the quality of early social‐emotional experiences (indicated by attachment security and maternal sensitivity) is related to infants’ monitoring of the interaction of the two abstract figures and explores the role of the available emotional information. Twelve‐month‐old infants were shown a series of animations in which two abstract figures, a larger “parent” and a smaller “baby” figure, first moved together and then became separated because the “baby” figure was unable to follow the “parent” figure. Upon and during the period of separation—while the two figures remained still—the sound of either a crying or a laughing baby could be heard. This period was followed by one of two possible responses from the “parent” figure, who either returned to the “baby” figure or left. We were particularly interested in the relative amounts of infant attention to the two figures. Our monitoring measure was thus the fixation duration ratio for the “parent” figure relative to the “baby” figure during the still separation part and during the response part of the animations.

We expected that attachment security and maternal sensitivity would be related to monitoring patterns. With regard to the direction of the expected differences in the fixation duration ratio, we argue that it is possible to entertain two opposing hypotheses. Based on their own early experiences with their primary caregiver, secure infants and infants with more sensitive mothers may regard the role and availability of a caregiver as more relevant or salient in their developing “working models”, and they may develop a stronger anticipation that a caregiver should react (promptly) when a child is in need. These prior expectations and the accompanying heightened interest in the behavior of the caregiver may bias the attention of secure infants and infants with more sensitive mothers to focus more on the “parent” figure during the observation of the animations. One hypothesis would thus be that secure infants and infants with more sensitive mothers will have a larger fixation duration ratio for the “parent” figure during the separation and response parts of the animation than insecure infants or infants with less sensitive mothers.

Alternatively, one could argue that based on their early experiences, secure infants and infants with more sensitive mothers would have confidence in the availability and the prompt response of the “parent” figure and they would therefore look relatively less to the “parent” figure compared to the insecure infants. Dickstein and her colleagues (Dickstein et al. 1984), for example, found that insecure‐resistant infants show more social referencing with their mother when they are in an unfamiliar situation. As both these hypotheses are theoretically plausible, we take an exploratory approach in testing these two possibilities and emphasize that both would support the idea that attachment security and maternal sensitivity are already associated with biased processing of social interactions in infancy. The fact that previous literature with older children and adults showed task‐dependent and mixed results concerning the specific nature of the social information processing bias (see Dykas and Cassidy 2011) further justifies our explorative approach.

Furthermore, we hypothesized that witnessing a distress situation (i.e., animations accompanied by the sound of a crying infant) may invoke attachment‐related representations and experiences more strongly than the observation of animations accompanied by the sound of laughter. We therefore expected to find an interaction between emotional sound (crying vs. laughter) and attachment security/maternal sensitivity, with larger differences between secure and insecure infants and between infants with more and less sensitive mothers for the animations that were accompanied by crying sounds compared to animations that were accompanied by laughter sounds.

In addition, based on Johnson et al.'s findings (2007, 2010), we explored whether the type of response would also lead to differences in the monitoring by secure and insecure infants or by infants with more and less sensitive mothers. We hypothesized that as an indication of the violation of infants’ expectations, the relative fixation ratio will be larger for secure infants and for infants with more sensitive mothers when they watch the “parent” figure leave, while the opposite pattern is expected during the observation of the return response of the “parent” figure.

The content of the internal working models of disorganized infants is assumed to be not fundamentally different from that of non‐disorganized infants’ in terms of expectations of caregiving behavior in regular social interactions without a traumatic component, as signs of disorganization are supposed to be triggered by infrequently occurring parental frightening or frightened behavior (Main and Hesse 1990; Madigan et al. 2006). There is, however, evidence for the influence of disorganization on attention bias in the processing of social threat and other negatively valenced stimuli by infants and adults (Atkinson et al. 2009; Peltola et al. 2015). Therefore, we tested whether differences in monitoring emerge not only related to attachment security but also in relation to attachment disorganization.

In sum, we explored the direct relation between attachment quality and maternal sensitivity, and monitoring processes during the observation of emotionally charged, abstract, third‐party social interactions. A growing body of evidence suggests a relation between attention biases and infant temperament (Kiel and Buss 2011; Pérez‐Edgar et al. 2011; Nakagawa and Sukigara 2012). For example, Nakagawa and Sukigara (2012) found that 12 months old infants whose parents reported more negative affectivity showed greater difficulty in disengaging their attention from negative social stimuli. There is also an ongoing debate about the role of temperament in the formation of attachment relationships (see Van IJzendoorn and Bakermans‐Kranenburg 2012). We therefore assessed fearful temperament in infants to control for its effect on the relation between attachment quality and maternal sensitivity on the one hand and monitoring processes on the other hand. Besides the relative fixation duration ratio for the “parent” figure, we also measured overall fixation (i.e., anywhere on the screen) during the animations to test whether the influence of attachment or maternal sensitivity is specific to infants’ attention allocation or is present in overall looking.

## Method

### Participants

Sixty healthy, full‐term 12‐month‐old infants (26 boys and 34 girls, mean age = 375.63 days, *SD* = 9.29 days, range = 354–396 days) and their mothers participated in the study. Families were recruited through direct mail, addresses were provided by the city council. The mothers were all the biological mothers of the infants except for one who was a foster mother (mean age = 34.07 years, *SD* = 4.48 years). In 89% of the families, both parents had the Dutch nationality and in remaining families one or the other parent had a European (7%), South American (2%) or African (2%) nationality. Using a 5‐point scale for education level (1: primary school, 2: vocational school, 3: secondary school, 4: postsecondary applied education, 5: university degree), the mean education level of the combined mother and father score was 4.08 (SD = 0.80, range: 1.5–5.0), which indicates a relatively highly educated sample. Due to not passing the inclusion criteria for all eye‐tracking measures, data from 55 infants were included in the analyses for the Separation segment and from 43 infants for the Response segment. The criteria for inclusion in these analyses are explained in detail in the Data Analysis section.

### Overall procedure

For all our participants the laboratory visit started with the eye‐tracking experiment during which the animations were presented to the infants (this lasted about 5 min). The eye‐tracking experiment was conducted first in order to assure that the infants were alert but not yet too stressed to sit relatively still in their mother's lap and that they could thus provide good quality measurement. This was followed by the Strange Situation Procedure that took place in a different laboratory room (this lasted a maximum of 21 min). We continued with three episodes from the Laboratory Assessment Temperament Battery (Lab‐TAB*,* Goldsmith and Rothbart 1999) to measure infants’ temperament. Then mother and infant participated in “break”, “competing demands” and “free play” episodes to assess maternal sensitivity (15 min in total) in the same laboratory room (see detailed description below). Finally, the mother was asked to participate in an eye‐tracker experiment, which is not part of this study. Infants received a gift and the mothers had their travel costs reimbursed. The study was approved by the Ethics Review Board of the Institute of Child and Education Studies at our university. Caregivers signed informed consent forms before participation.

### Eye‐tracker experiment

#### Stimuli

**Figure 1:**
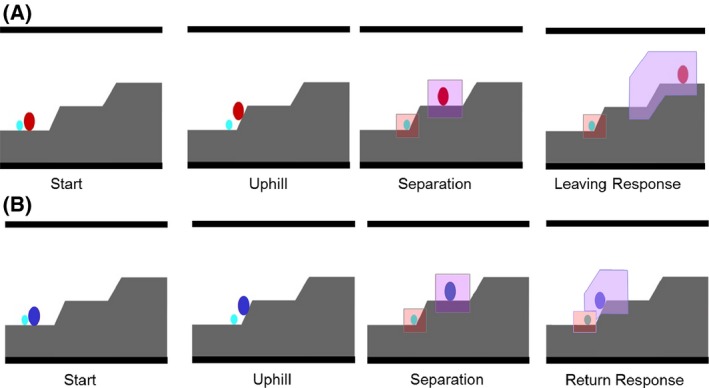
Frames from the Start, Uphill, Separation, Leaving Response (A), and Return Response segments (B) of the animation. Areas of interests (AOIs) for the two figures during the Separation and Response segments are shown (not visible to the infant).

Infants were presented with eight animations (34.5 × 25.5 cm with a resolution of 1270 by 924 pixels) involving two abstract characters: a larger “parent” (3.5 × 2.5 cm) and a smaller “baby” (2 × 1.5 cm) oval shape, see Figure 1, (see also Biro et al. 2014b for more details). Each animation started with the figures first moving together (Start segment, 2.6 sec). This was followed by the “parent” figure moving up a hill and stopping on the plateau while the “baby” figure was trying to go uphill but slipped back (Uphill segment, 2.1 sec). Upon separation the sound of a crying baby was played in half of the movies while the sound of a laughing baby was played in the other half (Separation segment, 11.0 sec). When the sound started, the “baby” figure expanded slightly (2 mm) and contracted three times together with a slight change in color (lasting 2.8 sec), giving the impression that it was the source of the sound. During the rest of the Separation segment the figures did not move. Following separation, in half of the movies (both for movies including crying and laughing sounds) the “parent” figure moved down the hill and ended up next to the “baby” figure (Return Response segment, 4.3 sec), whereas in the other half of the movies the “parent” figure moved further up a second hill and stayed on top of it (Leaving Response segment, 4.3 sec). The sounds of crying and laughter faded away during the last 2 sec. The color of the “parent” figure in the animations with the returning response was different from the one with the leaving response (blue vs. red), and counterbalanced across participants. The color of the “baby” figure was always light blue.

There were four order conditions to which the infants were randomly assigned. Two crying and two laughter movies were alternating, starting with either one or the other emotional type. In addition, the “parent” figure's response type alternated between every movie: the first four movies started with either Return or Leaving, and in the second four movies the response type alternated in the opposite order.

#### Procedure and apparatus

Infants’ eye‐movement patterns were recorded by a Tobii T120x eye‐tracker (Tobii technology AB, Sweden). Infants sat on their mothers’ laps in a curtained booth facing the 17” TFT monitor with the integrated eye‐tracker. The height of the chair and the position of the monitor were adjusted to establish a good eye‐tracking status (so infants’ eyes were 60 cm away from the monitor). Using Tobii Studio software, first a 5‐point infant calibration procedure was carried out. The presentation of the animations immediately followed the calibration. One of four different short attention‐getting movies was played in between the animations. Mothers were informed about the procedure, and were instructed not to talk and to try to keep the infants from moving or leaning. Mothers were wearing blinded sunglasses during the stimulus presentation.

#### Data analysis

A Tobii fixation filter was used with velocity and distance thresholds set to 35 pixels (see Gredebäck et al. 2012). Fixation measures were calculated using Tobii Studio software and further analyzed with SPSS. Two areas of interest (AOIs) covering the two figures were defined during the Separation segment: a Parent AOI (5.42% of the entire area) and a Baby AOI (2.19%), see Figure 1. During the Response segments the same Baby AOI was defined for the “baby” figure. For the “parent” figure, a Parent Going Away AOI (10.91%) was used in the Leaving Response that covered the area traversed by the “parent” figure while moving further up the hill, and a Parent Coming Back AOI (7.37%) was defined in the Return Response covering the path the “parent” figure took while descending the hill, see Figure 1. Note that the accuracy of the eye‐movement recordings did not allow for distinguishing fixations aimed at the “baby” and the “parent” figures when they were next to each other in the Return Response, therefore only the first period (2.3 sec) of both types of response segments was analyzed, during which the two figures in the Return Response were more than 1 cm away from each other.

Our area of interest related monitoring measure, the relative fixation duration ratio for the “parent” figure (the duration of fixations for the Parent AOI divided by the sum of the duration of fixations for the Baby AOI and Parent AOI), was calculated during the Separation and Response segments of each animation. The ratios were averaged separately for the two types of emotion signals during the Separation segment, and the ratios were averaged separately for emotion and response type in the Response segment. Because of the difference between the Leaving and the Return Response segments in the distance between the two figures, in the movement direction and in the size of the AOIs for the “parent” figure, the relative fixation ratios are not compared directly between the two types of response segments, as monitoring differences between the two types of responses could be explained by these inherent perceptual differences. Note, however, that the two types of responses can still be compared in terms of the differential influence of attachment quality and maternal sensitivity on infants’ relative fixation duration ratios in the AOIs. Furthermore, an overall looking measure, the total duration of fixations during the Separation and the Response segments in each animation was obtained and averaged in the same way as it has been done for the relative fixation ratio measure.

Infants were included in the analyses if they had eye‐tracking data in all four types of movies. This criterion resulted in the exclusion of four infants from the analysis of the Separation segment (*n *=* *56) and seven infants from the Response segment (*n *=* *53) for the overall looking measure. Furthermore, for the calculation of the relative monitoring measure infants had to have a fixation on either the Baby or Parent AOI in each type of movies. This criterion resulted in the exclusion of one more infant from the analysis of the Separation segment (*n *=* *55) and ten more infants from the Response segment (*n *=* *43). The excluded infants did not differ significantly from the included infants in their gender, mothers’ education, or in the observational measures (attachment quality, maternal sensitivity, or temperament), *P*s > 0.22.

### Attachment quality: Strange Situation Procedure

The Strange Situation Procedure (SSP, Ainsworth 1978), was used to measure the quality of infant‐parent attachment relationship. In short, the infant is introduced to an unfamiliar laboratory environment and a female stranger. The mother leaves the room twice and then returns to the room, leaving the infant alone for a short period, first with the stranger and then by her/himself. Attachment behavior during the two reunion episodes was coded according to the Ainsworth (1978) and Main and Solomon (1990) coding systems by two certified coders. One of the coders is a certified trainer in SSP coding and the other coder was trained by Alan Sroufe and Elizabeth Carlson. Both coders were blind to other information about the infants. On the basis of the ABC classification, we used the secure (B) vs. insecure (non‐B) distinction for the analysis. On the basis of the ABCD classification of the coders, we used the disorganized (D) vs. non‐disorganized (non‐D) distinction for the analysis. One third of the sessions (*n* = 20), which were randomly selected, were coded by both coders. Intercoder agreement for these cases was 75% (*κ *= 0.50) for B vs. non‐B and 85%, (*κ *= 0.69) for D vs. non‐D. Disagreements on the double‐coded reliability set were resolved by using the scores of the certified trainer. For the B vs. non‐B classification, 32 infants were securely attached and 28 were insecurely attached (8 avoidant and 20 resistant). With regard to the D vs. non‐D classification, 17 infants were classified as disorganized and the remaining 43 as not disorganized (27 secure, 3 avoidant, 13 resistant). The distribution of infants who were included in the eye‐tracking analysis for the Separation segment (*n* = 55) was 28 secure and 27 insecure, and 16 D and 39 non‐D. For the eye‐tracking analysis during the Response segment (*n* = 43), the distribution was 23 secure and 20 insecure, and 13 D and 30 non‐D.

### Maternal sensitivity assessment

Maternal sensitivity was measured during three episodes. During the *Break* (5 min) the mother and baby were offered refreshments. An infant chair for feeding and a standardized set of toys were also available. Next, in the *Competing demands task* (5 min) (Klinkman 1997) the mother was asked to fill out the Infant Characteristics Questionnaire (ICQ). The toys were taken away during this period. During *Free Play* (5 min) the mother was asked to play with the infant with a large set of toys that was provided. Maternal sensitivity was assessed using the 9‐point Ainsworth Sensitivity scale (Ainsworth et al. 1974) (1 = “highly insensitive” and 9 = “highly sensitive” mother). All three episodes were coded separately and the scores were then averaged to create an overall score for maternal sensitivity (Cronbach's alpha = 0.68). A trained researcher, blind to other information, coded all the sessions. A second, expert coder coded 15 participants. Intercoder reliability was adequate, the intra‐class correlation coefficient (single measure, absolute agreement) was 0.80 for the average score. Disagreements on the double‐coded reliability set were resolved by using the scores of the trained researcher who coded all the sessions. Average scores on maternal sensitivity ranged from 3.5 to 7.5 (M = 5.96, SD = 0.87). These scores were centered for the analyses.

### Temperament measure: observed fearfulness

The “Remote controlled spider” episode from the Lab‐TAB (Goldsmith and Rothbart 1999) was used to assess infants’ fearfulness. The infant was seated on the floor while the parent sat behind the child on a chair and was instructed not to interfere. We used a remote controlled car (instead of a spider) that was dressed with fur making its appearance unfamiliar to the infants. The car approached the infant, stopped for 10 sec and then retracted. This sequence was repeated three times unless the infant became too upset. At the end of the episode the infant was offered to touch the car if he/she wanted to. The episode was coded according to the guidelines (Goldsmith and Rothbart 1999). All raw scores were converted to z‐scores before composite scores were created. A fear composite score was derived from a trained coder's ratings on the intensity of facial fear (0–3), distress vocalization (0–5), bodily fear (0–3) and escape (0–3), *M *=* *0.00, *SD* = 0.73. A second coder coded 20 cases. Intercoder reliability was adequate, intra‐class correlation coefficient (single measure, absolute agreement) was 0.85 for the fear composite. Two additional episodes (the “Puppet game” and the “Attractive toy behind barrier”) were also administered to all infants in the same order, but these are not used in this study.

## Results

Preliminary analyses showed that infant gender, the color of the “parent” figure and the order of the presentation of the animations had no effect on the relative fixation duration ratio or on the overall fixation duration in the Separation and the Response segments, *P*s > 0.10. Furthermore, no significant correlations were found between attachment security (secure vs. insecure), attachment disorganization (disorganized vs. not‐disorganized), maternal sensitivity and fearfulness scores using bivariate Pearson correlations or chi‐square test in case of two categorical variables (*P*s > 0.17), except between the disorganized and security classifications (*χ* ^2^ = 5.45, df = 1, *P *=* *0.02, *n *=* *60, two‐tailed). The same pattern of correlations is present in the group of infants who had valid eye‐tracking data. Note that although the correlation between maternal sensitivity and attachment security was not significant (*r *=* *0.20, *P *=* *0.13, *n *=* *60), the effect size is in the range of what has been found meta‐analytically in nonclinical samples using the Strange Situation Procedure, *r *=* *0.22, *k *=* *30, *N *=* *1,666, 95% CI = 0.18–0.27, (De Wolff and Van IJzendoorn 1997).

### Areas of interest related monitoring measures

#### Attachment quality

**Figure 2:**
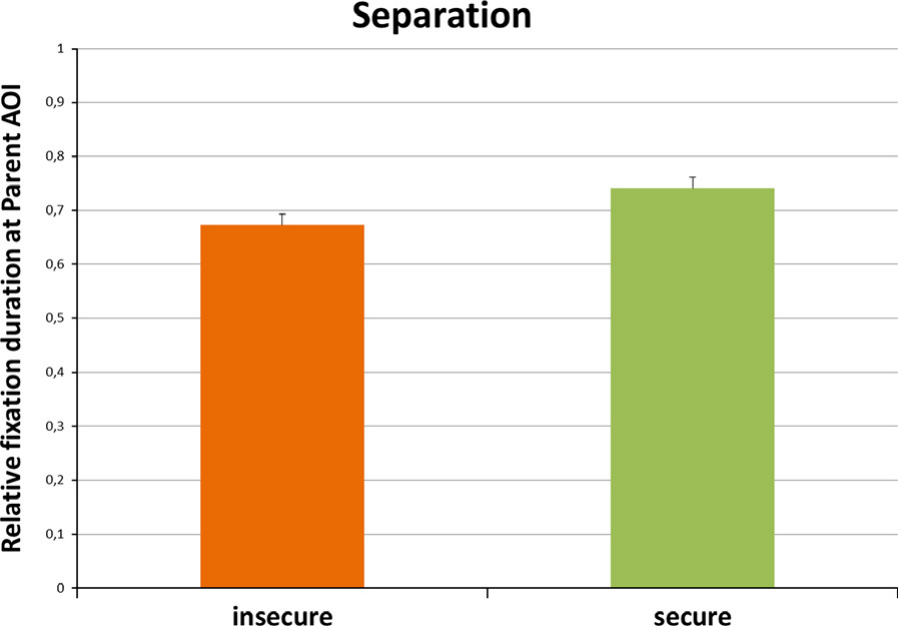
The mean relative fixation duration ratios with standard error for the Parent AOI during the Separation segment by the securely and insecurely attached infants (*P < 0.05).

A repeated measures ANOVA was carried out using the relative fixation duration ratio for the Parent AOI during the Separation segment with emotion (crying, laughter) as a within‐subject variable, and attachment security (insecure, secure infants) and disorganization (D, non‐D) as between‐subject variables. We found a main effect of security, *F*(1,51) = 5.09, *P *=* *0.03, $\eta_{p}^{2}$ = 0.09, and no interaction between security and emotion, *P *=* *0.35. Secure infants spent more time looking at the Parent AOI relative to the Baby AOI than insecure infants (see Figure 2). No main effect of disorganization nor interaction between disorganization and emotion was found for the Separation segment, *P*s > 0.09. When infants’ fearful temperament was included as a covariate in the analysis, the security effect remained significant, *F*(1,48) = 5.39, *P *=* *0.025, $\eta_{p}^{2}$ = 0.10, and no further changes in the pattern of results emerged.

A similar repeated measures ANOVA was carried out for the Response segment with the additional within‐subject variable of response type (Leaving, Return). We found no main or interaction effects of emotion, attachment security, disorganization, or interaction effects of response type, *P*s > 0.07. The inclusion of temperament as a covariate did not alter these results.

#### Maternal sensitivity

**Figure 3:**
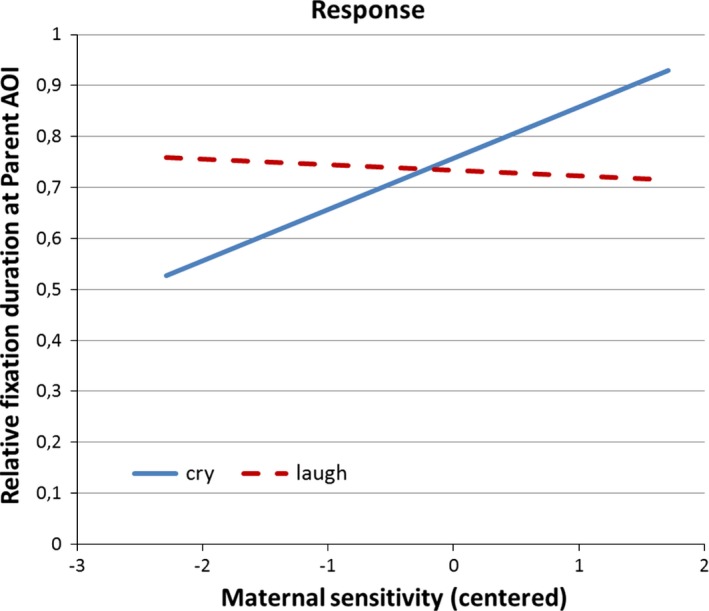
Association between relative fixation duration for the “Parent” AOI and maternal sensitivity in Response segment during the animations with crying and laughter sound.

Repeated measures ANOVAs were carried out using the relative fixation duration ratio for the Parent AOI separately for the Separation and the Response segments with emotion signal type and, in case of the Response segment, also with response type as within‐subject variables, and with maternal sensitivity as a continuous predictor. For the Separation segment no main or interaction effect of sensitivity was found, *P*s > 0.18 regardless of whether temperament was included as a covariate. For the Response segment the ANOVA revealed an interaction between emotion and sensitivity, *F*(1,41) = 13.24, *P *=* *0.001, $\eta_{p}^{2}$ = 0.24. Separate tests for the animations with the two types of emotion signal showed that sensitivity had an effect on the infants’ fixations when the crying sound was heard, *F*(1,41) = 10.53, *P *=* *0.002, $\eta_{p}^{2}$ = 0.20, but not when laughter was heard, *F*(1,41) = 0.14, *P *=* *0.71, $\eta_{p}^{2}$ = 0.003. This finding indicates that infants with more sensitive mothers looked longer at the “parent” figure relative to the “baby” figure when they heard the crying sound, see Figure 3. When infant fearful temperament was included as a covariate the interaction between sensitivity and emotion remained significant, *F*(1,39) = 11.83, *P *=* *0.001, $\eta_{p}^{2}$ = 0.23.

### Overall looking measures

To test whether infants’ overall attention to the animations (and not only their specific monitoring of the interacting characters) was influenced by the emotional signal, attachment security, disorganization or maternal sensitivity, similar repeated measure ANOVAs were carried out using the total fixation duration in both segments. No effects of attachment security, disorganized attachment or maternal sensitivity were found in any of the segments, *P*s > 0.13. However, we found a main effect of emotion both for the Separation segment, *F*(1,55) = 9.09, *P *=* *0.004, $\eta_{p}^{2}$ = 0.14, and for the Response segment, *F*(1,52) = 4.68, *P *=* *0.03, $\eta_{p}^{2}$ = 0.08, indicating that in both segments infants looked longer at the animation when they heard the crying sound compared to the laughter sound. Both effects remained significant when temperament was included as covariate or when attachment or sensitivity was not included in the analyses, *P*s < 0.04.

## Discussion

Attachment security was associated with the allocation of infants’ attention during the still separation part of the animation (i.e., after the “baby” figure was unable to follow the “parent” figure but before the “parent” figure reacted). In particular, we found that secure infants fixated longer at the “parent” figure relative to the “baby” figure than insecure infants during the separation accompanied by both infant crying and laughter sounds. Maternal sensitivity was related to monitoring during the response part of the movie (i.e., when the “parent” figure reacted). Infants of more sensitive mothers had a larger fixation ratio regardless of what the “parent” figure was doing (leaving or returning), but only when combined with infant crying sounds.

It is important to point out that the influence of attachment security and maternal sensitivity was specific to the attention allocation between the two interacting characters as there were no differences in the overall looking at the animations. Attachment and sensitivity therefore do not affect how much infants are generally interested in watching ongoing animated interactions. Furthermore, the associations of attachment security and maternal sensitivity with monitoring were present even when infant temperament was controlled for.

These findings therefore confirmed that early experience with the primary caregiver is related to attentional biases in social information processing. We put forward two hypotheses regarding the direction of monitoring differences. We argued that based on their experience, secure infants and infants with more sensitive mothers develop stronger expectations about the caregiver's availability and its prompt and appropriate response. We hypothesized that these expectations would either attract the attention of secure infants and infants with more sensitive mothers to the “parent” figure (as an indication of the importance and relevance of the “parent” figure while processing the interaction) or would focus the attention of the insecure infants and infants with less sensitivity mothers on the “parent” figure (due to their continued uncertainty about the availability of the caregiver). Our findings support the first hypothesis but future studies need to confirm whether this particular direction of attentional allocation bias is specific to the situation we have shown to infants or can be generalized to other observed parent–baby interactions. We speculate that because infants viewed abstract third‐party interactions in which they were not personally involved, it is less likely that the attention processes of insecure infants and infants with less sensitive mothers were driven by anxiety or by the lack of their ability to get quick reassurance (see Dickstein et al. 1984). Instead, we think that their attention processes were driven by their lower expectations about the importance of the role of a parent figure in resolving situations.

We expected that the monitoring differences would be more pronounced in the distress (crying) situation because it is more likely to invoke attachment‐related representations. The fixation ratio differences were, however, only specific to distress in case of maternal sensitivity in the response part of the animation. We do not have a straightforward explanation for this pattern, if anything, the opposite pattern would have been expected since responsive and sensitive caregiving occurs in infants’ everyday life also in non‐stressful situations, whereas attachment behavior and related representations are more likely to be tied to comfort seeking in distress situations. Bowlby (1969) however, argued that a wide range of situations in which the availability of the caregiver was relevant, also without extreme distress, would activate and shape the attachment system. In the laughter separations, the “baby” figure was also unable to follow the “parent” figure, and that might explain why in secure infants it also elicited longer fixations at the “parent” figure relative to the “baby” figure. With regard to the segment specific effects of our two measures, we can only speculate that the separation segment can be viewed as more analogous to the episode in the SSP situation in which the infant is left alone and hence that attachment security may be more strongly related to differences in infants’ expectations when “parental” behavior has not yet been observed. Maternal sensitivity may be more strongly linked to infants’ interest in the attachment figure's ongoing behavior, which is observed during the response segment. Future research testing infants’ monitoring in different comfort‐ or help‐related social contexts could reveal whether the distress specificity and segment specificity of the sensitivity vs. attachment security effect is a robust phenomenon.

Considering alternative or additional explanations for the monitoring differences, one might argue that since the animations were repeated eight times, infants may have learned that it is the “parent” figure who would move eventually. The larger fixation ratio for the “parent” figure during the separation in secure infants may indicate such learning. Our preliminary data analysis, however, revealed that all infants showed an increase in their fixation ratio across the repetition of the animations, regardless of their attachment quality, indicating that all infants learned to expect that the “parent” figure will respond after the separation. The security effect cannot therefore be explained solely by a general learning advantage of secure infants.

Another way to interpret the distress‐specific fixation ratio difference in the response segment is that it may reflect the difficulty of infants with less sensitive mothers with disengaging attention from the source of distress (the “baby” figure). The literature is inconclusive whether such an explanation is plausible. Forssman et al. (2014) showed that maternal stress and depression are associated with heightened infant attention to social signals of fear. At the same time, Peltola et al. (2015) suggest the opposite, at least for the influence of attachment: insecure infants tend to direct their attention away from negative stimuli.

One can also entertain the possibility that the differences in both segments were caused by simple perceptual preferences such as secure infants and infants with more sensitive mothers like to look at bigger figures more than insecure infants and infants with less sensitive mothers. While such low level perceptual preferences cannot be ruled out, it is not a parsimonious explanation to account for the fixation differences because (1) it cannot explain the emotional signal specificity that we found, (2) it would be hard to explain how differences in early social‐emotional experiences would lead to such perceptual preferences, and (3) we know of no other studies finding perceptual preference differences related to infant attachment.

Overall, while this study may not allow us to precisely tease apart all different cognitive or attentional processes that could underlie the monitoring differences, we showed that security and maternal sensitivity were associated with specific monitoring and, importantly, that the direction of the effects were the same; they both increased the attention toward the “parent” figure.

Besides security of attachment we also explored the influence of disorganized attachment and found no difference between disorganized and non‐disorganized infant groups. This suggests that disorganization is not related to infants’ monitoring of abstract third‐party interactions, or at least not in the particular context that we showed to the infants. As a link between disorganized infant attachment and frightening/frightened caregiver behavior has been reported (Main and Hesse 1990; Schuengel et al. 1999), it might be possible that an observed interaction that contains elements of threat would elicit monitoring patterns that are specific to disorganized infants.

Attachment and maternal sensitivity were not associated with overall looking, but we did find an emotion effect on overall looking in both Separation and Response segments. This partly replicates our previous finding (Biro et al. 2014b) and thus supports a general negativity bias often found in emotional information processing research with infants (e.g., Vaish et al. 2008). Negative signals such as crying are hypothesized to elicit more attention because they carry more information or because they have a general arousal effect. While general arousal may have elicited more overall looking in the distress animation in our study, it cannot account for the specific monitoring pattern differences.

We did not find differences in the monitoring or in the overall looking measure between the two types of “parent” response with regard to attachment security or maternal sensitivity. To interpret the lack of difference in relation to the findings of Johnson et al. (2007, 2010), we have to note that our design is not a violation of expectation paradigm with habituation and test phases during which looking times are measured *after* different outcomes have been presented. We used a within‐subject design in which all four types of animations are alternatingly and repeatedly shown to the infants for a fixed duration and monitoring was measured during the *ongoing* interaction. Our data during the response part can therefore not be directly compared to Johnson's data or be viewed as failing to replicate Johnson's findings. Importantly, we did show that monitoring differences were present between secure and insecure infants during the separation part of the animation, and this finding complements Johnson et al.'s results.

In sum, in this study, we showed that infant attachment security and maternal sensitivity were associated with 12‐month‐old infants’ monitoring strategies while they were watching abstractly depicted social interactions. These findings support the hypothesis that early social‐emotional experiences with the primary caregiver bias infants’ social‐cognitive information processing. We suggest that these biases are markers of infants’ developing “internal working models” (Bowlby 1969; Bretherton and Munholland 2008; Dykas and Cassidy 2011). In the context of attachment theory, internal working models (IWMs) are assumed to serve various functions (as discussed in Dykas and Cassidy 2011) including storing attachment‐related knowledge, predicting interactions with attachment figures, providing information about self and—most relevant to this study—influencing social information processing. Two modes of strategies have been proposed about how IWMs operate depending on whether the processing of information could lead to potential psychological pain (Bowlby, 1973; Bretherton and Munholland 2008; Dykas and Cassidy 2011). A defensive strategy suppresses information to avoid pain and a schematic strategy leads to a biased processing of (non‐painful) information that is consistent with previously obtained attachment‐related knowledge. In this study, individual differences in monitoring most likely reflect a schematic processing strategy: attention allocation differences related to attachment security and maternal sensitivity were consistent with infants’ knowledge, expectations, or familiarity (e.g., insecure infants and infants with less sensitive mothers attending less to the “parent” figure or turning more toward the distressed “baby figure”). The lack of difference in overall looking further suggests that no suppression of social information occurred while infants were passively viewing the abstract animations.

Attachment theory also predicts that the content and organization of the IWMs of avoidant and resistant infants within the insecure classification should be different and it is thus expected that these two groups would show different patterns in the biases of social‐cognitive information processing (Dykas and Cassidy 2011). Empirical evidence confirms this by showing that in some (but not all) circumstances avoidant/dismissive individuals show a greater tendency to suppress information while resistant/preoccupied individuals show more biased processing of negative information (Main et al. 1985; Kirsh and Cassidy 1997; Mikulincer et al. 2002; Carnelley et al. 2007; Rholes et al. 2007). Our sample size was too small to address this question with confidence. Nevertheless, our preliminary explorative analysis did not reveal differences between the two insecure groups. Consistent with this finding, in the Johnson et al.'s studies (2007, 2010, Experiment 1) no differences emerged in the looking times between avoidant and resistant infants. However, in a slightly modified version of the original experiment, Johnson et al. (2010, Experiment 2) found that when infants witnessed that the “baby” figure either approached or stayed away from the “parent” figure after a distressed separation and partial reunion, only avoidant infants (and not resistant or secure infants) looked longer at the outcome of the “baby” figure being reunited with the “parent” figure. Future research is thus needed to test whether differences between insecure subgroups in attentional processes during ongoing monitoring may also be sensitive to specific contexts.

In terms of the causal pathway, we proposed that social‐cognitive information biases are the products of differences in early social‐emotional experiences between infants. Others suggest the possibility that inherent, independent attentional or genetic biases shape the way individuals subjectively experience the environment, and that these biases are, at least in part, responsible for differences in attachment and attachment‐related representations (Johnson and Chen 2011; Peltola et al. 2015). Alternatively, another third factor could also be a common cause leading to differences in both attention and attachment security. These causal directions are generally difficult to tease apart and this is particularly the case when measures are taken concurrently. The fact that in our study monitoring was associated with both maternal sensitivity and attachment supports the first option. Future studies using longitudinal designs could investigate the causal direction of these associations much more precisely, particularly if not only sensitivity and attachment but also social‐cognitive processing biases are measured at different ages.

A further possible future direction is motivated by Song et al.'s (2015) study in which they showed that children's drawings became more affiliative when they were primed with observed third‐party ostracism videos. In our study, the order of the assessment for eye‐tracking and SSP was fixed and we think it is unlikely that the prior viewing of animations could have had a major impact on the classification of the infants as secure or insecure. Nonetheless, whether the infants’ behavior during the SSP can be influenced by observational priming, or whether the monitoring patterns can be influenced by prior personal attachment‐related experience, are interesting questions.

In closing, our main findings show that sensitive caregiving and infants’ attachment security are associated with differences in the focus of infants’ attention. We propose that these attention biases reflect individual differences in infants’ developing internal working models that shape the way infants perceive the social world around them.

## Conflict of Interest

None declared.
